# Comparison of Needle Knife versus Scissors Forceps for Colorectal Endoscopic Submucosal Dissection: A Prospective Randomized Study

**DOI:** 10.3390/jcm12062329

**Published:** 2023-03-16

**Authors:** Tatsuo Yachida, Hideki Kobara, Kazuhiro Kozuka, Kaho Nakatani, Naoya Tada, Takanori Matsui, Taiga Chiyo, Nobuya Kobayashi, Shintaro Fujihara, Noriko Nishiyama, Akihiro Kondo, Yasuhisa Ando, Keiichi Okano, Wakako Nonaka, Kaori Ishikawa, Hisashi Masugata, Tsutomu Masaki

**Affiliations:** 1 Department of Gastroenterology and Neurology, Faculty of Medicine, Kagawa University, Kita 761-0793, Kagawa, Japan; 2 Department of General Internal Medicine, Faculty of Medicine, Kagawa University, Kita 761-0793, Kagawa, Japan; 3 Department of Gastroenterological Surgery, Faculty of Medicine, Kagawa University, Kita 761-0793, Kagawa, Japan

**Keywords:** endoscopic knife, colorectal endoscopic submucosal dissection

## Abstract

Background and Aim: To evaluate the efficacy and safety of a grasping-type knife, called Clutch Cutter (CC), for colorectal endoscopic submucosal dissection (C-ESD). Methods: This was a randomized prospective study. Patients who underwent C-ESD for colorectal neoplasms >20 mm and <50 mm in size were enrolled, dividing into two groups: ESD using needle type of dual knife alone (D-group) and circumferential incision using dual knife followed by submucosal dissection using CC (CC-group). The primary outcome was the self-completion rate. The secondary outcomes were intraoperative complication rate, procedure time, and en bloc resection rate. Results: A total of 45 patients were allocated to the D-group and 43 to the CC-group were allocated. The self-completion rate was higher in the CC-group (87% [39/45] vs. 98% [42/43]). All of the six patients with an incomplete procedure in the D-group were completely resected with CC use. The intraoperative complication rate was not significant in either group (D vs. CC: 2% vs. 0%). The mean procedure time was significantly shorter in the D-group than that in the CC-group (62.0 vs. 81.1 min; *p* = 0.0036). The en bloc resection rate was 100% in the D-group and 98% in the CC-group. Conclusions: While dual knife use is superior to CC in terms of time efficiency, the use of CC may be a safe and efficacious option for achieving complete C-ESD.

## 1. Introduction

Endoscopic submucosal dissection (ESD), which enables en bloc resection and detailed pathological diagnosis, has provided an excellent outcome for curative resection of gastrointestinal (GI) neoplasms at early stage [1,2]. However, compared to other organs (esophagus and stomach), colorectal ESD (C-ESD) is considered more difficult due to the anatomical features. The colorectum has the anatomical characteristics of a thin wall and rich folds and bends, which causes an intraoperative perforation with the higher rate. Accordingly, C-ESD requires sophisticated techniques even by experienced specialists. Although the knives dedicated for ESD have been recently developed to facilitate C-ESD, the choice of these knives remains unclear [3]. A needle type of knife has been commonly used for C-ESD. The main concern is intraoperative perforation, especially when operators use the knife perpendicularly to the muscle layer. Consequently, its usage may be associated with a high rate of adverse events (AEs), longer procedure time, and the need for more skillful endoscopic techniques [4]. During submucosal dissection, conventional knives, such as the needle knife and ball tip type of IT knife (Olympus, Tokyo, Japan), gently push the knife into the submucosal tissue and cut it with electrosurgical current. This cutting manner has the potential of electrosurgical burning to the muscle layer, leading to increased risk of intra/postoperative AEs [5]. To reduce the AEs risk related to ESD with a conventional knife, a grasping-type scissor (Clutch Cutter [CC]; Fujifilm Medical, Tokyo, Japan), which can accurately grasp, pull, coagulate, and/or incise the targeted tissue, was developed [5]. In detail, CC can simultaneously achieve hemostasis and cut vessels, hold the targeted tissue, and dissect safely at the confrontation part of the muscle layer. The approach is similar to that of laparoscopic surgery, and seems acceptable for any performers. There are two elements of manipulations for tumor resection: the endoscopic element and the forceps element. If the endoscopic element could be eliminated in resections, manipulations would be simplified and colorectal ESD could be made universal not only for expert but also for less experienced endoscopists. The CC, developed by Akahoshi et al. [6], was commercialized for use in June 2010. Previous reports showed the safety and time efficiency of CC for difficult cases with high perforation risk in esophageal and gastric ESD [7,8,9,10,11]. Although its usefulness in C-ESD has been reported [12], the superiority of CC over existing other knives remains fully unknown.

This study aimed to examine the strengths of the grasping-type knife by comparing a needle knife versus a grasping-type knife for C-ESD.

## 2. Methods

This study was a randomized controlled trial conducted at a single academic medical center. The present study was approved by the Clinical Ethics Committee of Kagawa University Hospital (H24-075) and was registered in the University Hospital Medical Information Network Clinical Trials Registry (UMIN No. 000010551). All patients provided written informed consent.

### 2.1. Patient Selection

**Figure 1:**
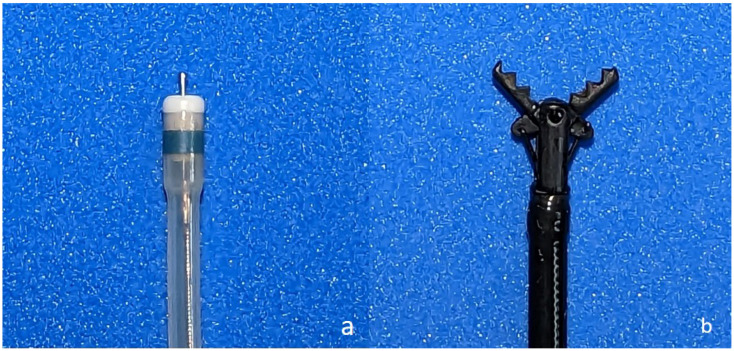
(**a**) Dual knife (KD-650Q; Olympus, Tokyo, Japan); (**b**) Clutch Cutter (DP2618DT-35-; Fujifilm Medical, Tokyo, Japan).

Patients who underwent ESD for superficial colorectal superficial neoplasms between January 2011 and April 2016 were enrolled. All patients were randomly assigned to undergo C-ESD using either the dual knife (Olympus, Tokyo, Japan) (D-group) (Figure 1a) or dual knife plus grasper scissors (CC) (CC-group) (Figure 1b). Randomization codes were packed into sealed opaque envelopes by blinded individuals to ensure concealment of allocation. The characteristics of lesions, including morphology, tumor size, location, and invasion depth suspected by pit pattern diagnosis, were preoperatively evaluated by colonoscopes with magnifying function (CF-H260AI, PCF-Q260AZI, Olympus, Tokyo, Japan). The extent and depth of the tumors were carefully assessed. Macroscopic types were classified according to the Paris classification and Kudo’s classification as follows: type O-I (protruded) and two subtypes of laterally spreading tumors (LSTs). The two subtypes of LSTs were either granular (LST-G) or non-granular (LST-NG). The inclusion criteria were colorectal adenoma or early carcinoma with 2 ≤ lesion size < 5 cm according to Japanese guidelines [2]. The exclusion criteria were as follows: patients with preoperative diagnosis of submucosal (SM) deep invasion, a blood clotting disorder or organ failure, those who did not provide informed consent, and lesions located in areas where the endoscopist was unable to hold the scope during preoperative colonoscopic examination.

### 2.2. ESD Procedures

Patients were generally administered intravenous midazolam (0.05 mg/kg) and pethidine (50 mg) prior to the procedures. Colonoscopes with a water jet function (PCF- Q260J or Q260AI, Olympus, Tokyo, Japan) were introduced. A transparent cap that was longer at the tip (Elastic Touch F-030, Top Corporation, Tokyo, Japan) was applied. VIO 300D (ERBE Elektromedizin, Tiibingen, Germany) was used as the power source for electrical cutting and coagulation. The setting was EndoCut mode effect 2, duration 3, coagulation mode: 60 W, effect 5, swift coagulation; 30 W, effect 4.

ESD was performed after placing several dots around the tumor with a margin of approximately 5 mm. Half of the hyaluronic acid solution (MucoUp, Boston Scientific, Marlborough, MA, USA) and concentrated glycerin was injected into the submucosa using a 25-gauge endoscopic injection needle (SureLIFTER, Boston Scientific, Marlborough, MA, USA) just outside the margin of the tumor to elevate the lesion. A small incision and circumferential incision were made using a dual knife in both groups. Submucosal excision was performed using a dual knife or Clutch Cutter, according to the allocation. To stop bleeding or prevent hemorrhage before vessel cutting, hemostatic forceps Coagrasper (Olympus, Tokyo, Japan) were used in the D-group, while Clutch Cutter was used in the CC-group. When a continuation of ESD was difficult, we used the CC to prioritize en bloc resection of neoplasms. In the D-group, the operators could change from dual knife to Clutch Cutter during mucosal incision and submucosal dissection when they encountered one or more of the following technical difficulties: (i) severe fibrosis in the submucosal layer; (ii) inability to incise and dissect using the dual knife, with difficulty controlling the endoscope as a result of paradoxical or respiratory movement, with the operators judging it dangerous to continue using the dual knife; and (iii) inability to access the submucosal layer, because of inadequate lifting of the submucosa after the additional submucosal injection of hyaluronate sodium. On the other hand, in the CC-group, the operators could use a variety of devices when the continuation of ESD was difficult for technical reasons.

After the neoplasm was removed, the resected specimen was retrieved using net forceps. The post-ESD ulcer was carefully observed, and exposed vessels were coagulated with a Coagrasper in D-group or CC in CC-group using a soft coagulation mode to avoid delayed hemorrhage. All procedures were performed by three experienced endoscopists (T.Y., H.K., and N.N.) who had successfully performed more than 200 gastric ESDs and more than 50 C-ESDs.

### 2.3. Examination Protocol

The patients were hospitalized for 6 days after C-ESD. On postoperative day 1, blood examinations were performed to check for complications, and then the diet was started.

### 2.4. Data Analysis and Outcome Measures

Procedural details were recorded prospectively in a database. Collected data included the following: patient sex and age; macroscopic type: protruded lesion or LST-G (HG: homogenous type), LST-G (NM: nodular mixed type), LST-NG (FE: flat elevated type) or LST-NG (PD: pseudo-depressed type); tumor distribution and size. The characteristics of the lesions in the CC-group were compared with those in the D-group. The primary outcome was the self-completion rate. The secondary outcomes were intraoperative complication rate, procedure time of technically successful cases, and en bloc resection rate. The self-completion was defined as both the technical completion and the achievement of en bloc resection. The incompletion of procedure was defined as when the procedure could not be completed using the designated knives in each group. The procedure time was defined as the duration between the beginning of circumferential incision and the completion of en bloc resection. All ESD specimens were fixed in 10% formalin and evaluated pathologically. Pathological diagnoses were made by a highly experienced clinical pathologist and were categorized according to the Japanese Classification of Colorectal Carcinoma proposed by the Japanese Society for Cancer of the Colon and Rectum.

### 2.5. Sample Size Calculation

Prior to the start of this study, the self-completion rate of C-ESD using a dual knife was calculated as 85% based on our previous database. We assumed that the yield of technical completion with D-group would be 80% as an intermediate value. Therefore, we hypothesized that the CC-group would raise by about 15% the technical completion yield compared to the D-group. Assuming a 5% significance level and a statistical power of 80% using a two-sided equivalence (α = 0.05 (2-sided), power (1−β) = 0.8), power analysis indicated that the total sample size was 80, requiring 40 cases in each group. Considering some excluded cases, 43 cases in each group was set as the target sample size.

### 2.6. Statistical Analysis

Continuous variables were compared using the Student’s *t*-test. Categorical variables were compared using χ^2^-tests or Fisher’s exact tests, as appropriate. *p* < 0.05 was considered statistically significant. Statistical analyses were performed using the JMP 15.1 software package for Windows (SAS Institute, Cary, NC, USA).

## 3. Results

**Figure 2:**
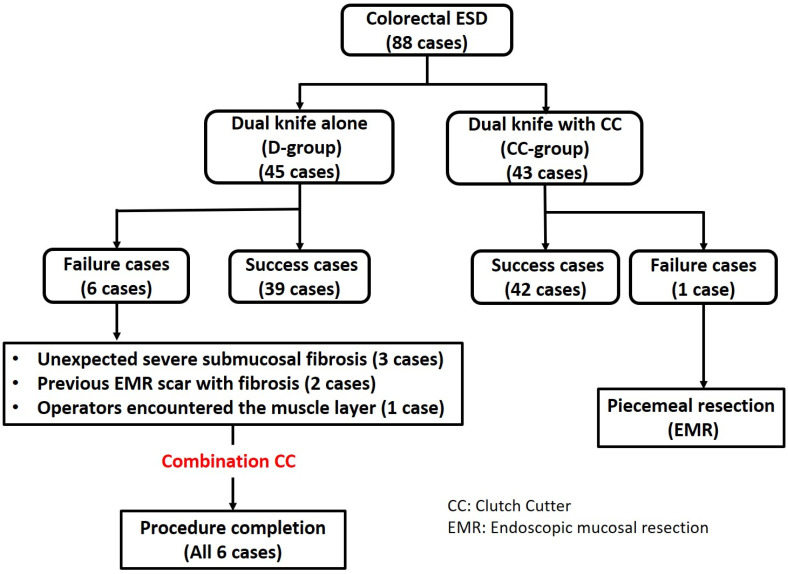
Flowchart of patient enrollment.

A total of 88 patients (men: 55, women: 33; median age: 69 years, range: 26–88 years) were enrolled, and all patients (45 patients in the D-group and 43 in the CC-group) completed the study. The flowchart of patient enrollment is shown in Figure 2. The clinical backgrounds and their detailed clinicopathological data are shown in Table 1. The clinicopathological values were not significantly different between the two groups in terms of sex, age, macroscopic type, distribution, resection size, and tumor size (*p* > 0.05).

### Outcome Results

**Figure 3:**
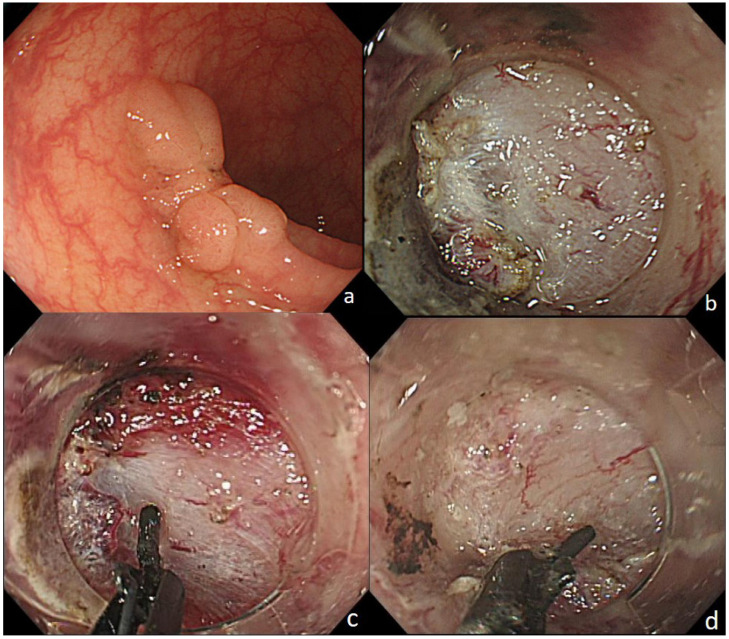
(**a**) The lesion is located in the sigmoid colon, the tumor size is 25 mm, and the macroscopic type is LST-G (nodular mixed type). (**b**) The muscular layer is faced and sufficient lift by local injection is not possible. (**c**) CC could grasp the target site and safely separate it. (**d**) In the final excision scene, the visual field facing the muscular layer could be reliably separated.

The results of outcomes are summarized in Table 2. The self-completion rate was higher in the CC-group (87% [39/45] vs. 98% [42/43]) (*p* = 0.11). All of the six patients with incomplete procedure in the D-group were completely resected using CC. These patients comprised three patients with unexpected severe submucosal fibrosis, two patients with previous EMR scar, and one patient with specimen located in the transverse colon where operators encountered the muscle layer during submucosal dissection (Table 3). Case 1, Case 2, and Case 3 were sigmoid colon and Rs, located in a flexure, with fibrosis under the lesion. A dual knife, which is a needle type of knife, was considered to have an extremely high risk of perforation; therefore, the CC was used. Case 4 and Case 5 were post-EMR recurrent lesions with hard fibrosis owing to the scar and the lesion could not be safely resected with a dual knife, and hence CC was used. Case 6 was located in the flexure of the transverse colon, and in any position, a dual knife would have encountered the muscle layer. Therefore, the risk of vertical perforation was high and CC was required. A representative patient (Case 2) rescued by CC is shown in Figure 3. In the CC-group, one patient had an unsuccessful procedure of piecemeal snare resection because the lesion located in the rectum-sigmoid was difficult to approach the lesion from the tangential direction even with all scope manipulations and positional changes. The intraoperative complication rate was not significant in either group (D vs. CC: 2% vs. 0%) (*p* = 1.00). The mean procedure time was significantly shorter in the D-group, excluding six failure cases, than the CC-group, excluding one failure case (mean ± SD, 62.0 ± 4.4 vs. 81.1 ± 4.6 min; *p* = 0.0036). The en bloc resection rate was 100% in the D-group and 98% in the CC-group (*p* = 1.00).

## 4. Discussion

This was the first study that compared CC with conventional dual knife in C-ESD. We found that the self-completion rate tended to be higher with the combined use of CC, and incomplete C-ESD cases in the dual knife alone could be completed with the additional use of CC.

ESD is a recent minimally invasive surgery to achieve successful en bloc resection of early stage gastrointestinal neoplasia. To date, most endoscopists are willing to develop devices that allow ESD to be quicker, safer and more effective. A variety of high-frequency electric knives have been developed for ESD, such as the dual knife, hook knife (Olympus, Tokyo, Japan) [13], ball tip type of IT knife nano (Olympus, Tokyo, Japan) [14,15], flush knife (Fujifilm Medical, Tokyo, Japan) [16], flex needle knife (Olympus, Tokyo, Japan) [17], bipolar needle knife (B-knife) (Zeon Medical, Tokyo, Japan) [18], and grasping-type of SB knife (Sumitomo Bakelite, Tokyo, Japan) [19]. These knives are mainly classified into a needle type knife, a ball tip type knife, and a grasping-type knife. The dual knife, traditionally produced in 2009, is a popular needle type knife. However, the usage of dual knife alone has the potential risks of intraoperative perforation and bleeding in the following situations: where operators use the knife perpendicularly to the muscle layer, lesions with severe submucosal fibrosis or rich vessels, difficult scoping maneuvers because of respiratory movement. A grasping-type knife including CC can be easily handled in a simple manner of ‘grasp’ and ‘cut’. Moreover, most knives are designed alone for cutting or dissection, but are not favorable for vessel shielding and hemostasis during ESD. The grasping-type knife provides a multifunction of cutting and hemostatic procedures without changing the devices. Therefore, we compared the efficacy of a dual knife and CC in the present study.

CC enables the secure fixation and lift-up by grasping and has a thin serrated cutting edge and an insulated coating on the outer side of the forceps. These characteristics facilitate grasping of the targeted tissue and concentrate electrosurgical current energy at the blade to avoid burning the surrounding tissue.

The self-completion is a key element for complete ESD. A comparative study of grasping-type scissor forceps and insulated-tip knife for ESD dissection of early gastric cancer has been conducted [11], and CC was reported to be safe and effective for ESD in the esophagus, stomach, and colon [5]. In a recent randomized controlled trial that compared another scissor-type forcep, the SB Jr knife with flush needle knife in C-ESD, the self-completion rate for trainees was significantly higher in the SB Jr group than that in the Flush group (67% vs. 39%, *p* = 0.01), while the procedure time did not differ significantly between the two groups (59 vs. 51 min; *p* = 0.14) [20]. In contrast, this study included experienced operators. The present study also revealed that the self-completion rate was higher in the CC-group than D-group (86% [39/45] vs. 98% [42/43]). Intraoperative perforation occurred in one case of the D-group, although it was not significant in either group (D vs. CC: 2% vs. 0%). One case of perforation occurred near the anastomosis after sigmoidectomy, and the scope was difficult to control, resulting in a micro-perforation at the time of circumferential incision with the dual knife. Previous reports using CC in the colon have also reported a 0% perforation rate [5,7,21].

The grasping step has three safe effects: fixation effect, lift-up effect, and compression effect. The grasping step prior to electrosurgical incision allows the fixation of the device to the targeted tissue to avoid unintentional incision. The scissor-type knife can be handled easily, just like handling the biopsy forceps, even in an unstable position, because the operator simply grasps the target tissue and cuts it without endoscopic movements, even at a remote distance; hence, a scissor-type knife might reduce the risk of perforation. This enables the operator to overcome difficult situations, such as an unstable approach to the lesion, and inability to access the submucosal layer for dissection. Thus, CC can be a safe knife for submucosal dissection. Therefore, in the present study, additional CC use rescued all the six patients with incomplete procedure in the D-group as these cases had one or more of the above-mentioned situations, limiting the use of a dual knife.

The disadvantage of grasping-type knives is that they are time consuming. Previous studies have shown that C-ESD using grasping-type knives requires more than twice the mean procedure time compared with needle-knives [5,21]. The reported mean procedure time of C-ESD using a grasping-type scissors forceps was 155 min [21]. Meanwhile, the mean operating time of ESD using knife devices ranges from 61 to 110 min [22,23,24,25,26,27]. Our study also showed that the mean procedure time was significantly shorter in the D-group than that in the CC-group (mean, 62.0 vs. 81.1 min; *p* = 0.0036). In this study, the procedure time was considered excluding the six cases that had difficulty using the dual knife alone, and therefore may have been shorter in the D-group. In addition, the reasons for this are the need for the confirmation three steps: fixation, lift-up, and resection step for grasping-type knives. In contrast, the dual knife seems to be superior because it can make a single straight incision in areas where the submucosa can be raised sufficiently to enable safe dissection.

Several reports using CC for colorectum ESD showed high rates of en bloc resection (97–100%) [12]. In our study, the en bloc resection rate was 100% in the D-group and 98% in the CC-group (*p* = 1.00). This is comparable with results of other studies using other devices (84–91.5%) [22,23,24,25,26,27].

## 5. Limitations

This study has several limitations. First, this was a single-center study. Second, since three experienced operators participated in this study, the self-completion rate and the procedure time for trainees could not be clarified. Third, the decision to change the knives by the operators was subjective, which could have caused bias when comparing the self-completion rate between the two groups. Fourth, because a circumferential incision using dual knife plus submucosal dissection using CC was applied in the CC-group, the efficacy of CC was evaluated only in sessions of submucosal dissection. Thus, the combination of the dual knife and CC may not be cost-effective, and to clarify the cost-effectiveness and efficacy of CC in C-ESD, a comparison of a dual knife vs. CC should be further carried out.

Furthermore, CC can be used to grasp the targeted tissue again if the grasped site is inadequate prior to electrosurgical cutting and these operations are simple and easy, similar to the bite biopsy technique.

## 6. Conclusions

While dual knife use is superior to CC in terms of time efficiency, the use of CC may be a safe and efficacious option for completing C-ESD.

## Figures and Tables

**Table 1:** Clinicopathological characteristics and outcomes of the D-group and CC-group.

	D-Group (45 Cases)	CC-Group (43 Cases)	*p*
Sex (male/female)	28/17	27/16	* p * = 0.96 *
Median age, years (range)	70 (41–84)	68 (26–88)	* p * = 0.79 **
Macroscopic type			NS
Protruded lesion	7	8	
LST-G (HG)	4	6	
LST-G (NM)	17	18	
LST-NG (FE)	12	6	
LST-NG (PD)	5	5	
Distribution			NS
Cecum	4	5	
Ascending colon	7	13	
Transvers colon	11	7	
Descending colon	3	1	
Sigmoid colon	11	7	
Rectum	9	10	
Median resected size (range, mm)	35 (21–60)	35 (18–60)	* p * = 0.36 **
Median tumor size (range, mm)	25 (20–49)	25 (20–50)	*p* = 0.07 **

D-group, dual knife group; CC-group, Clutch Cutter group; LST-G (HG), laterally spreading tumor, granular and homogenous type; LST-G (NM), laterally spreading tumor, granular and nodular mixed type; LST-NG (FE), laterally spreading tumor, nongranular and flat elevated type; LST-NG(PD), laterally spreading tumor, nongranular and pseudo-depressed type; * χ^2^-tests; ** Student’s *t*-test; NS, Not Significant.

**Table 2:** Outcome results.

	D-Group (n = 45)	CC-Group (n = 43)	*p*
Self-completion rate	87% (39)	98% (42)	*p* = 0.11 *
Complication rate			
Perforation	2% (1)	0% (0)	*p* = 1.00 *
Delayed bleeding	0% (0)	5% (2)	*p* = 0.24 *
Mean procedure time, min (±SD)	62.0 (±4.4)	81.1 (±4.6)	*p* = 0.0036 **
(excluding failure cases)	(39 cases)	(42 cases)	
En bloc resection rate	100% (45)	98% (42)	*p* = 1.00 *

D-group, dual knife group; CC-group, Clutch Cutter group; * Fisher’s exact test; ** Student’s *t*-test.

**Table 3:** Investigation of six failure cases in the D-group.

	Case1 (71 y.o. M)	Case2 (81 y.o. M)	Case3 (43 y.o. M)	Case4 (70 y.o. F)	Case5 (69 y.o. M)	Case6 (76 y.o. F)
Macroscopic type	LST-NG (FE)	LST-G (NM)	0-Isp	LST-G (NM)	0-Is	LST-G (NM)
Procedure time (min)	140	100	117	70	100	111
Resected size (mm)	35	25	35	45	21	35
Tumor size (mm)	28	20	30	40	20	30
Distribution	Sigmoid	Sigmoid	Rs	Rb-P	Transverse	Transverse
Characteristic	Fibrosis	Fibrosis	Fibrosis	EMR scar	EMR scar	Encountered the muscle layer
Depth	Adenoma	Adenoma	sm2 (1000 μm)	m	Adenoma	Adenoma
Retroflex view	-	-	+	+	-	-
Procedure completion	+	+	+	+	+	+

D-group, dual knife group: M, Male; F, Female; LST-G(HG), laterally spreading tumor, granular and homogenous type; LST-G (NM), laterally spreading tumor, granular and nodular mixed type; LST-NG(FE), laterally spreading tumor, nongranular and flat elevated type; LST-NG(PD), laterally spreading tumor, nongranular and pseudo-depressed type.; Rs, rectosigmoid; Rb; rectum below peritoneal reflection; P, proctodeum; sm, submucosa; m, mucosa

## Data Availability

Data are not publicly available so as to protect personal data and maintain medical confidentiality.
